# Cultural inclusion and corporate sustainability: evidence from food culture and corporate total factor productivity in China

**DOI:** 10.1057/s41599-023-01649-3

**Published:** 2023-04-11

**Authors:** Guangfan Sun, Xin Lin, Junyi Chen, Nuo Xu, Ping Xiong, Hanqi Li

**Affiliations:** 1 School of Business, Renmin University of China, Beijing, China; 2 School of Literature and Law, Zhengzhou Technology and Business University, Zhengzhou, China; 3 School of Mathematics and Computer, YuZhang Normal University, Nanchang, China; 4 Department of Geography & Anthropology, Louisiana State University, Baton Rouge, LA USA

**Keywords:** Finance, Business and management, Cultural and media studies, Geography

## Abstract

This article constructed a food taste deviation index using text analysis based on food culture as a measure of cultural inclusion. Cultural inclusion was related to corporate total factor productivity, aiming to investigate whether the cultural inclusion affects corporate sustainability. The findings showed uneven distribution of cultural inclusion in various areas of China, contributing to the unbalanced sustainable development of firms in different regions, as reflected by the higher total factor productivity of firms located in regions with higher cultural inclusion. A test using mountainous undulation as an instrumental variable verified the causal relationship between cultural inclusion and firm sustainability. Besides, a regression discontinuity design was employed to mitigate the impacts of the bidirectional causality. Moreover, a high level of cultural inclusion in top managers’ hometown significantly improved corporate sustainability, suggesting that executives can transmit cultural inclusion in society. In addition, firms in more inclusive regions are more motivated to increase their social responsibility to staff as a way to promote their total factor productivity, suggesting that cultural inclusion promotes firm sustainability by increasing firms’ care for staff. This article also found that cultural inclusion has heterogeneous effects across firms of different levels of industry competition, and different levels of analyst attention. The research reveals the impact of cultural inclusion on the real economy and help academics dissect the informal institutional reasons behind the sustainable development of firms in different geographies from a new perspective, contributing to the promotion of economic sustainability at the regional level and related policy formulation.

## Introduction

The interdisciplinary study of culture and finance has been at the forefront of research in economics and finance in recent years (Lei et al., 2022). There is ample evidence that culture has a profound impact in shaping national financial systems at the macro level and business sustainable development at a microscopic level (Guiso et al., 2006). Since culture has been recognized as an important factor influencing financial development, the influence of geoculture on corporate governance patterns and business behavior has been recognized by numerous researchers (Chen et al., 2014; Guiso et al., 2006; Guiso et al., 2009). However, research on how inclusive cultural characteristics at the geographic level affect corporate sustainability has never been studied.

Inclusiveness, as a concept that advocates equality of opportunity, plays a key role in maintaining social equity and improving the well-being of people (Shore et al., 2011; Shore et al., 2018). Since suffering from COVID-19, economic development around the world has been negatively influenced. Faced with the negative externalities caused by COVID-19, the sustainable development of firms is facing a serious situation. Countries have formulated corresponding regulations accordingly to improve the external environment and help firms to resume production. China, in particular, as a country with a large population and the world’s largest developing country, faces greater challenges in achieving corporate sustainability with the deregulation of COVID-19. However, there is a lack of academic studies how inclusion affects corporate finance. The current relevant research has focused on the impact of inclusion within corporate internal organization (Shore et al., 2011). How the inclusion of the external environment affects the sustainable development of firms is an important topic to be discussed. Therefore, we focused on whether cultural inclusion, such an important geographical cultural characteristic, could have a significant impact on the corporate sustainability.

We chose China as our research scenario based on the following advantages unique to China. The traditional Chinese culture is deeply influenced by clans, and social norms such as geoculture is even more important to Chinese than official legislation. In a country like China, where formal institutions are becoming increasingly sophisticated but still lacking, informal institutions such as geoculture have a non-negligible impact on ecological economic and sustainable business (Allen et al., 2005). Moreover, China’s complex and diverse geographical location and natural environment have created unique geoculture in different regions. Large-scale cross-regional population movements within China have further contributed to the formation of a diverse cultural environment. Therefore, China’s rich regional cultural differences make it an ideal scenario for studying how geoculture affects corporate sustainability (Lei et al., 2022).

We are committed to filling the gap on cultural inclusion and corporate finance through exploring how cultural inclusion influences firm sustainability. In particular, we investigated whether cultural inclusion would enhance the corporate humanistic care to employees and improve corporate total factor productivity (*TFP*). *TFP* of firms is an indicator to comprehensively measure the efficiency of firm development and is used in an increasing number of research to evaluate corporate sustainability (Miao and Wang, 2012; Olley and Pakes, 1996; Petrin, 2003; Sun et al., 2022). Using Chinese listed companies as our research sample, we constructed a cultural inclusion index based on food culture. We found that cultural inclusion significantly increases firm total factor productivity, and this correlation is more obvious among firms in higher industry competition, and firms with higher analyst attention. Further analysis showed that firms in more inclusive regions typically increased their level of care for staff and thus promoted sustainable business growth, demonstrating that the humanistic spirit of firms is the mechanism of action for cultural inclusion. We also performed instrumental variable regressions using mountain undulation to alleviate potential endogeneity issues and conduct a series of robustness tests to verify our findings. Moreover, we employed a regression discontinuity design (RDD) to mitigate the impacts of the bidirectional causality. We further investigated how cultural inclusion spreads through society. This issue is pivotal because of not only enriching our intuitions about the formation and transformation of cultural inclusion but also alleviating endogenous concerns. We found top managers can bring cultural values from their home regions and help disseminate these values through corporate leadership.

The potential contributions to our article include: first, it expands the emerging literature on culture and finance, which currently focuses on themes such as sin culture, regional humanism, and collectivism (Boubakri and Schoar, 2016; Gu et al., 2019; Wang et al., 2022). This paper constructed a cultural inclusion index based on food culture and investigated the impact of cultural inclusion on the corporate total factor productivity. To our knowledge, our research might be the first to suggest that cultural inclusion affects corporate sustainability. The contribution of this paper also lies in empirically testing the positive effect of food culture on promoting sustainable business development. Second, this paper extends the basic research of the influence corresponding to informal institutions toward corporate productivity. Current academic research on the factors influencing corporate *TFP* has focused on corporate characteristics and formal institutions (Ahuja and Katila, 2001; Ekholm et al., 2012; Hsieh and Klenow, 2009; Riverabatiz and Romer, 1991). This paper explored antecedents of corporate *TFP* based on the perspective of informal institutions, enriching the research results that study culture and total factor productivity of firms. Third, this paper explored the transmission path and intrinsic mechanism of cultural inclusion. The empirical results suggest the humanistic effect of cultural inclusion, providing a plausible explanation for the influence channel of cultural inclusion. Finally, this paper explored how informal institutions affect the corporate sustainability in different areas based on cultural inclusion, helping to explore the antecedents of social norms behind corporate sustainability and has some implications for the formulation of sustainable development policies in different countries.

Our remaining arrangement is as follows: chapter “Research analysis and assumption” states our research analysis and assumption, detailing the relationship between food culture and cultural inclusion; chapter “Data and variables construction” focuses on our dataset; chapter “Empirical results” presents our basic regression, endogeneity treatment, regression discontinuity design, and robustness tests; chapter “Further analysis” includes transmission exploration, mechanism analysis and cross-sectional inspection; chapter “Conclusion and implications” reports our conclusion and implications.

## Research analysis and assumption

### Definition of cultural inclusion

Studies have been conducted to reveal that inclusion at the organizational level has a significant impact on business development. Shore et al. (2011) relied on the theory of optimal differentiation to explain the definition of organizational inclusion as the level of respect that employees perceive from their organizational system. Inclusiveness is expressed in the degree to which people experience a sense of belonging and the extent to which the organization embraces their own uniqueness. Inclusiveness, when used as a regional cultural characteristic, reflects the degree of acceptance and respect shown by local residents when dealing with individuals, groups, things, etc., that have different attitudes or ways of doing things than their own (Levine et al., 2005). In general, inclusiveness at the territorial level refers specifically to the extent to which the region respects and embraces external cultures, which would involve full understanding and fair treatment of people with different customs and cultures (Algan et al., 2016; Lowes et al., 2015).

### Food culture and cultural inclusion

Cultural inclusion is a multidimensional concept that is influenced by many aspects such as nature, social norms, and cultural identity, etc. How to accurately measure cultural inclusion is the key issue in this paper. Current academic research has focused on inclusive economic growth and how inclusive leadership within firms affects business development (Groysberg and Slind, 2012; Kashyap et al., 2021; Rajan and Zingales, 1998; Shore et al., 2011), and there is a dearth of studies corresponding to the impact of inclusion in the external environment on sustainable business development. We referred to Lei et al. (2022), who used the local diversity of dialects as a proxy of cultural diversity. Lei et al. (2022) used dialects as carriers of geoculture because each dialect contains specific cultural patterns and ways of thinking. Therefore, they argued that language is a better proxy of culture. Similarly, we used the inclusion of food culture as a proxy for cultural inclusion, because food is a necessity for human society and is important in maintaining interpersonal relationships, characterizing local cultural connotations, as well as promoting the process of sustainable local economic development (Cook, 2006; Cook, 2008; Cook et al., 2011).

We now describe specifically the reasons why the inclusion of food culture can serve as a good proxy variable for cultural inclusion. In terms of natural attributes, food can be seen as a product of human interaction with nature. As a natural product, food is largely governed by geographical features (e.g., weather, rivers, mountains, etc.), and thus, food culture has a very distinct regional character. In the dimension of social attributes, food has become an important link in social relations. From the rituals between nations to the table culture, food plays an important role as a link to maintain relationships. In ancient China, powerful people often shared food to strengthen ties with their subordinates; in modern times, Chinese people still eat and gather to strengthen their bonds with family and friends. In many countries, sharing food and eating together is also understood as a process in which encounters occur, especially among different ethnic cultures, where food may act as a mediator (Chesters, 2007; Sun et al., 2022). In the dimension of cultural attributes, food is an important vehicle for strengthening local emotions. A large body of literature has shown that food culture affects the group’s sense of local identity and local attachment in many ways (Cappellini and Yen, 2013; Chuck et al., 2017; Cross and Gilly, 2014; Kikon, 2021). After a long history of precipitation and solidification, certain unique tastes have gradually been symbolized as local cultural symbols; for example, sweetness is often regarded as an important food culture symbol in places like Zhejiang and Jiangsu, while umami is representative of the food cultures of Fujian and Guangdong. This phenomenon of constructing local identity through gustatory memory is also related to geo-relations to some extent, reflecting the role of geography in human’s deep-rooted emotional activities. To sum up, food culture is a product of human-nature interaction, as well as a bond of social relations and a carrier of local culture and emotion (Cappellini and Yen, 2013; Chang et al., 2010; Lo Monaco and Bonetto, 2019). Thus, the inclusion of food culture may be a good proxy for cultural inclusion (Cook, 2006; Cook, 2008; Cook et al., 2011).

Food culture carries deep regional emotions, and the unique taste of local food is regarded as the indication of its local food culture (Chang et al., 2010; Li et al., 2021; Trachootham et al., 2018). Specifically, “spicy” is often regarded as the symbol of Sichuan and Hunan diet, as well as “umami” is usually linked to Guangdong and Fujian dish (Zhu et al., 2018). These regional tastes describe food culture through the inheritance of taste memory, which builds the basis of food culture (Chang et al., 2010; Li et al., 2021; Trachootham et al., 2018). Thus, Our main measure of inclusion in the external environment is the extent to which a region’s food tastes deviate from the tastes of its native cuisine, with the connotation that the region will be strongly resistant to external food cultures to prevent assimilation (Chang et al., 2010; Cook, 2006; Cook, 2008; Cook et al., 2011; Karababa and Ger, 2011). Therefore, a higher degree of deviation of food tastes from indigenous cuisines could represent a more far-reaching impact of cultural inclusion in the region.

### Assumption

Theoretical and empirical analyses so far have shown that the sustainable development of firms is not only related to their own behavior such as export behavior (Criscuolo et al., 2010), resource allocation (Hsieh and Klenow, 2009), corporate mergers and acquisitions (Ahuja and Katila, 2001), but also influenced by external factors such as trade liberalization (Amiti and Konings, 2007), economic integration (Riverabatiz and Romer, 1991), international competitive pressures (Ekholm et al., 2012), and tariff changes (Fan et al., 2018). Geoculture as a social norm has a profound impact on economic development and financial market improvement, and may have an impact on the development of a firm in various ways, such as improving the external environment of the firm or optimizing the internal governance of the firm.

Numerous studies have shown that geoculture (e.g., sin culture, regional humanism, collectivism, etc.) plays an extremely important role in the sustainable development, sustainable operation and sustainable governance of firms (Boubakri and Schoar 2016; Gu et al., 2019; Wang et al., 2022). Influenced by the local culture, the management of the firm will show homogeneous cultural characteristics in their thoughts and actions, which will be further reflected in the corporate sustainable development, such as organizational planning and governance decisions (Chen et al., 2014; Lei et al., 2022). The cultural inclusion we are concerned with is an explicitly geoculture that reflects a level of respect and acceptance of something external and different from oneself. Cultural inclusion may influence a firm’s growth by affecting its external environment and internal governance.

The existing literature suggests that individuals with different cultural backgrounds differ significantly in terms of their upbringing and values. These differences can lead to different results in the analysis of knowledge. Knowledge that is considered commercially valuable by some individuals may not be considered valuable by others. Thus, the evaluation of the commercial value of knowledge may differ among individuals (Audretsch et al., 2010). For a given knowledge, the richer the group of different cultural backgrounds within the region, the greater the difference in the valuation of the knowledge. Knowledge that is not considered commercially valuable by locals may be highly valuable to outsiders. Thus, homogeneous groups may overlook some specific development opportunities, while the existence of differentiated groups can enhance the use of available knowledge and improve regional development. An inclusive external environment can precisely enhance local respect and acceptance of groups with different cultural backgrounds, thus creating a more humanistic atmosphere and more likely to attract external talents to contribute to the sustainable development of local firms (Van Knippenberg and Schippers, 2007). Thus, the integration of an external environment may facilitate different ways of commercializing the use of knowledge by local firms. External individuals can combine existing knowledge in new ways to contribute to the sustainable development of local firms (Desrochers and Leppala, 2011).

In addition, more inclusive regions are likely to have greater tolerance and respect for business. Firms have more incentive to choose the path that best suits them and are less worried about being criticized by external rating agencies and the public. Firms also do not have to cater too much to the needs of external stakeholders, which can lead to resource misallocation and inhibit their growth (Hsieh and Klenow, 2009). Therefore, the external environment itself may be more humane and inclusive. Moreover, an inclusive external environment may further alleviate the financing constraints of firms. Adequate inclusion and respect for firms may make it more possible for firms to obtain sufficient sources from external stakeholders for achieving sustainability (Campello et al., 2010). In summary, cultural inclusion may create a good external environment suitable for sustainable development of firms.

On the other hand, an inclusive external environment may lead to harmony within the firm. In more inclusive regions, firms are also more likely to recruit talent with a combination of backgrounds. People with multiple backgrounds may see problems from different perspectives thus generating more solutions to problems and contributing to the corporate sustainable growth. Firms may be influenced by external environment inclusion, thus become more tolerant of the diversity of their staff’ approaches to problem solving (Qian, 2013). Firms may also be influenced by an inclusive external environment to give more humanistic care to their staff, thus inspiring organizational citizenship behavior (Hancock et al., 2013; Paillé et al., 2014), which in turn leads to a positive internal corporate culture and promotes sustainable development (Van Knippenberg and Schippers, 2007). In addition, an inclusive external environment may lead to more tolerant and respectful qualities among stakeholders within the firm. For example, the presence of inclusion may enhance the humanism of the firm’s major shareholders, thereby inhibiting the hollowing out of minority shareholders, thereby alleviating the firm’s financing constraints and ultimately promoting sustainable corporate growth (Kim et al., 2011). In summary, we propose the following assumption.

#### Assumption 1a: Cultural inclusion can promote corporate sustainable development

However, different culture backgrounds are likely to result in conflicts and contradictions, which may be harmful to corporate sustainability. In culturally inclusive regions, groups may exhibit more complex and diverse cultural backgrounds. From an intra-firm perspective, the complex cultural backgrounds of groups may lead to cultural identity barriers (i.e., identity threat and identity fragmentation) and the creation of cross-cultural barriers. A diversity of knowledge may generate negative impacts on managerial decisions, which in turn affects the knowledge sharing and integration among team members, ultimately having a negative impact on the corporate sustainability (Leung and Wang, 2015). From an external perspective, the excessive complexity of external cultures reduces the ability of individuals to effectively integrate knowledge from different cultures, thereby inhibiting corporate sustainability (Chua, 2013). Therefore, we propose the following competing assumption.

#### Assumption 1b: Cultural inclusion can inhibit corporate sustainable development

Having discussed the relationship between cultural inclusion and corporate sustainability, we now explore how cultural inclusion spreads through society. CEO plays an important role in making decisions on corporate operational matters. This decision-making power allows CEOs to influence corporate decisions based on their own personality traits (Bertrand and Javalgi, 2003). Thus, CEOs may be able to make decisions that are consistent with their local culture and values through their leadership. When CEOs themselves are markedly culturally inclusive, they have a willingness to use their leadership to homogenously influence corporate governance behaviors, thereby increasing the impact of local cultural inclusion on corporate sustainability. Conversely, when CEOs’ personal preferences for inclusiveness are weaker than the culturally inclusive characteristics of the firm’s location, they may prefer to make decisions with reference to their own internal underlying cultural norms. This may lead to an increase in cultural conflicts in corporate decision-making processes and diminish the impact of local cultural inclusion on corporate sustainability. Therefore, we propose the following assumption:

#### Assumption 2: Significant cultural inclusion characteristics of CEO’s birthplace can strengthen the link between local cultural inclusion and corporate sustainability

Based on the above analysis, cultural inclusion may have a positive or negative impact on corporate sustainability. Therefore, we need to explore what kind of relationship exists between cultural inclusion and corporate sustainability through empirical tests.

## Data and variables construction

### Data description

We used a web crawler approach in August 2017 to obtain recipe information from “Meishijie” (http://www.meishij.net/), the largest recipe information website in China (“Meishijie” classifies “Chinese cuisine” into 20 cuisines, including Hunan, Anhui, and Hubei cuisines, according to the origin of the cuisines). We quantified the taste of a dish based on the frequency of seasonings used in the recipe (e.g., the Hunan dish “stir-fried pork with chili” uses seasonings such as chili, cooking oil, salt, soy sauce, chicken essence, etc.) (Li et al., 2021). Specifically, we classified tastes of food into seven dimensions: “spicy”, “sweet”, “salty”, “sour”, “umami”, “pungent” and “oily” (Li et al., 2021; Running et al., 2015; Ikeda, 2002). We used text analysis to assign each component of a dish to corresponding tastes to obtain taste quantitative value for each dish. Each component can be labeled as 0 to multiple tastes. For example, “ketchup” was labelled as “sour”, “sweet” and “oily” at the same time. We obtained the quantitative value of taste (Table 1) for each dish by equal weighted average of all dishes within the cuisine.

**Table 1:** Quantitative tastes of cuisines.

Sequence	Note	Cuisines	Sour	Sweet	Spicy	Umami	Salty	Pungent	Oily
1	Sixteen cuisines included in both recipes and PC data	Hunan	0.63	0.32	1.63	0.15	0.67	1.32	0.82
2		Guangdong	0.12	0.48	1.28	0.13	0.45	1.01	0.54
3		Qingzhen	0.38	0.61	1.68	0.28	0.94	2.01	0.92
4		Sichuan	0.95	0.54	1.75	0.27	0.51	2.23	0.72
5		Dongbei	0.26	0.43	1.61	0.25	0.64	1.34	0.68
6		Gangtai	0.34	0.78	1.66	0.15	0.6	1.79	0.84
7		Beijing	0.11	0.58	1.26	0.21	0.57	1.26	0.68
8		Shandong	0.16	0.36	1.24	0.18	0.57	1.14	0.68
9		Hubei	0.26	0.48	1.59	0.22	0.85	2.35	0.89
10		Shanghai	0.06	0.64	1.43	0.19	0.91	1.38	0.97
11		Anhui	0.13	0.6	1.18	0.17	0.69	1.16	0.77
12		Fujian	0.10	0.47	1.13	0.17	0.83	1.14	0.74
13		Jiangsu	0.09	0.51	1.26	0.15	0.58	1.17	0.69
14		Xibei	0.34	0.46	1.58	0.21	0.92	2.01	0.91
15		Yungui	0.66	0.52	1.68	0.25	0.74	1.90	0.92
16		Zhejiang	0.09	0.47	1.26	0.16	0.7	1.09	0.74
17	Only four cuisines included in recipes	Henan	0.17	0.53	1.43	0.21	1.01	1.61	0.91
18		Jiangxi	0.59	0.25	1.24	0.16	0.77	1.48	0.76
19		Shanxi	0.19	0.43	1.43	0.27	0.75	2.22	1.03
20		Guangxi	0.39	0.53	1.91	0.35	1.01	1.84	0.69

Note: “Sour” is an important food flavor. The most commonly used acid in food is acetic acid (the main component of vinegar). “Sweet” is a basic sense of taste, referring to the taste of sugar and honey, which is a popular sense of taste. In many cultures around the world, sweetness symbolizes a beautiful feeling. “Umami” is a complex and mellow feeling of food and a very important taste that reflects the taste of dishes. In Asia, monosodium glutamate is a very popular condiment, which can increase the umami taste of food. “Salty” is a mental sensory experience that a person distributes to the tongue, usually primarily from the salt that gives the tastebuds a boost. “Oily” usually comes from oil-rich foods, such as cooking oils and salad dressings. “Spicy” and “pungent” are not two names for the same flavor. “Spicy” refers to the flavor of ingredients from chili peppers, while “pungent” refers to the flavor of ingredients other than chili peppers, such as cinnamon, star anise and cumin, which have a pungent taste.

**Fig. 1:**
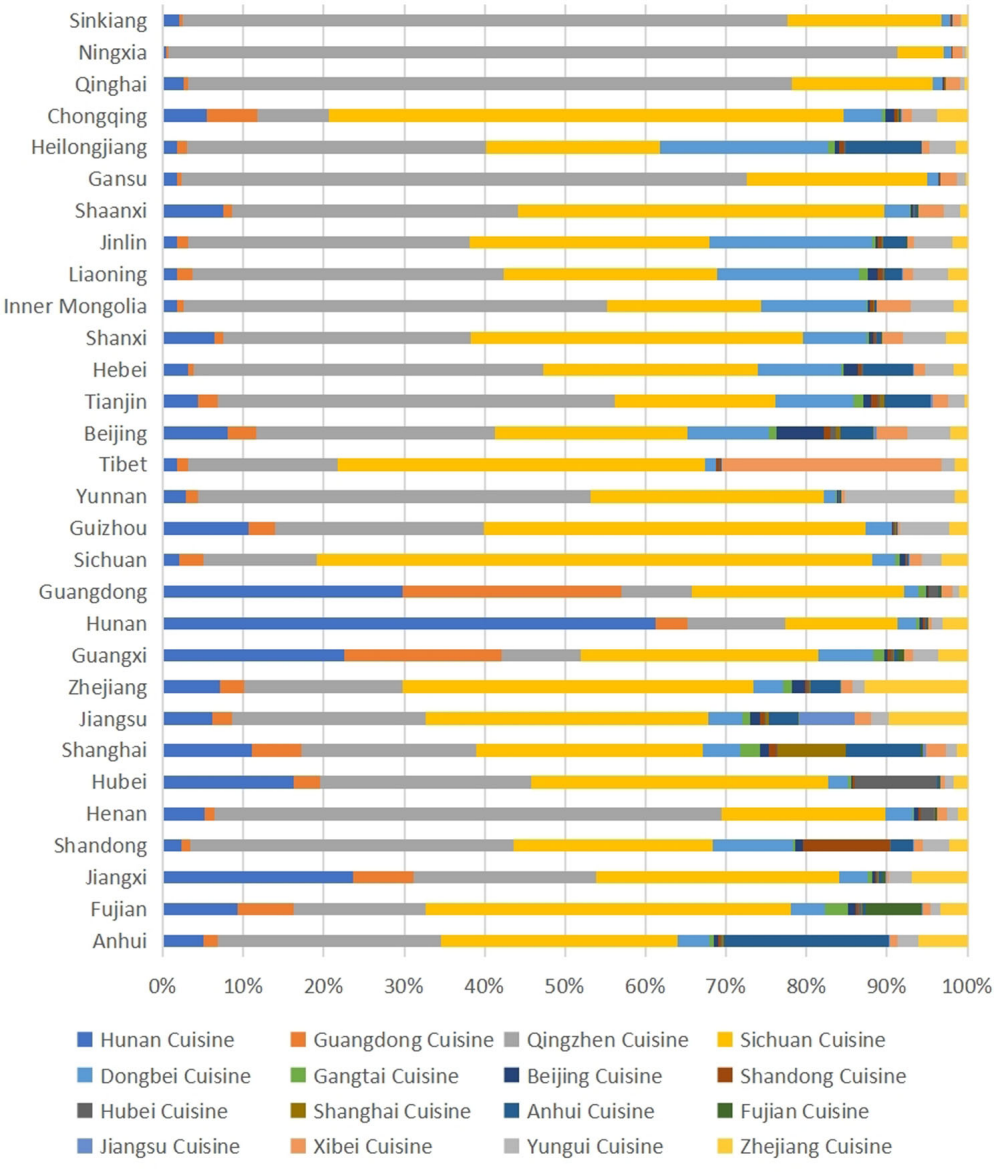
Percentages of various types of *PC* in China’s provincial units. *PC* is the position of catering. Each *PC* has its own cuisine, our study excludes Taiwan, Macao, Hongkong & Hainan.

Next, to quantify the food tastes of each provincial administrative unit in China, we crawled the position of catering (PC) data from Amap (https://www.amap.com/). The PC dataset contains over 7 million restaurant data, including detailed address and business information. We used the text analysis method to identify the restaurant’s corresponding cuisine from the operation information, and a total of 16 cuisines were identified, as shown in Table 1. We finally identified a total of 383,321 valid PC, the distribution of which is shown in Fig. 1.

We counted the number of PC for each type of cuisine in each province as a proportion of the total effective PC. Then, we calculated the quantitative value of each taste for each provincial administrative unit by combining the tastes of the cuisines in Table 1. Taking the “salty” flavors of Beijing as an example, we calculated the proportions of 16 PC among all valid PC in Beijing. Then, we used these proportions as weights to weight the quantitative values of “salty” flavors for each cuisine to obtain the quantitative values of “salty” flavors in Beijing.

We obtained data on financial and executive characteristics from CSMAR and collected data on geographic development from the National Bureau of Statistics of China. We then merged the corporate information with regional information. The sample period we chose is from 2010 to 2019, including10,140 firm-year samples and 2118 firms of A-share market, and excluding data from the ST category, PT category, financial and real estate sectors.

### Variable description

Our dependent variable was corporate total factor productivity (*TFP*). *TFP* is a variable to comprehensively evaluate the development efficiency and is widely used to evaluate the corporate sustainability (Miao and Wang, 2012; Petern, 2003; Sun et al., 2022). We used LP-semiparametric approach to account for *TFP* and take logarithm (*TFP_LP*) (Petrin, 2003).

Each provincial administrative unit in China has developed its own local cuisine. For example, “Sichuan cuisine” is the local cuisine of Sichuan and Chongqing, “Dongbei cuisine” is the local cuisine of Northeast China (i.e., Heilongjiang, Harbin, and Jilin), and “Xibei cuisine” is the local cuisine of Northwest China, etc. The cultural inclusion index (*C_Inclu*) was constructed by the deviation of the quantified taste values of provincial units from the quantified taste values of local cuisines. Using Beijing as an example, we subtracted the “salty” value of “Beijing cuisine” from the “salty” value of Beijing. Then we divided this value by the “salty” value of “Beijing cuisine”. We took the absolute value of the calculation result to get the “salty” taste deviation of Beijing. We calculated the deviation values of the seven tastes in turn and took their average values to obtain the overall degree of deviation, i.e., the cultural inclusion index (*C_Inclu*). The specific formula is as follows, where _*pr*_*p*_ represents the taste value of province *p*, _*diet*_*p*_ represents the local cuisine taste value of province *p*, and *C*_*Inclu*_*p*_ represents the cultural inclusion index of province *p*.

1 $$D_{spicy_p} = \left| {\frac{{spicy_{pr_p} - spicy_{diet_p}}}{{spicy_{diet_p}}}} \right|$$

2 $$D_{sweet_p} = \left| {\frac{{sweet_{pr_p} - sweet_{diet_p}}}{{sweet_{diet_p}}}} \right|$$

3 $$D_{salty_p} = \left| {\frac{{salty_{pr_p} - salty_{diet_p}}}{{salty_{diet_p}}}} \right|$$

4 $$D_{sour_p} = \left| {\frac{{sour_{pr_p} - sour_{diet_p}}}{{sour_{diet_p}}}} \right|$$

5 $$D_{umami_p} = \left| {\frac{{umami_{pr_p} - umami_{diet_p}}}{{umami_{diet_p}}}} \right|$$

6 $$D_{pungent_p} = \left| {\frac{{pungent_{pr_p} - pungent_{diet_p}}}{{pungent_{diet_p}}}} \right|$$

7 $$D_{oily_p} = \left| {\frac{{oily_{pr_p} - oily_{diet_p}}}{{oily_{diet_p}}}} \right|$$

8 $$C\_Inclu_p = \frac{{D_{spicy_p} + D_{sweet_p} + D_{salty_p} + D_{sour_p} + D_{umami_p} + D_{pungent_p} + D_{oily_p}}}{7}$$

**Fig. 2:**
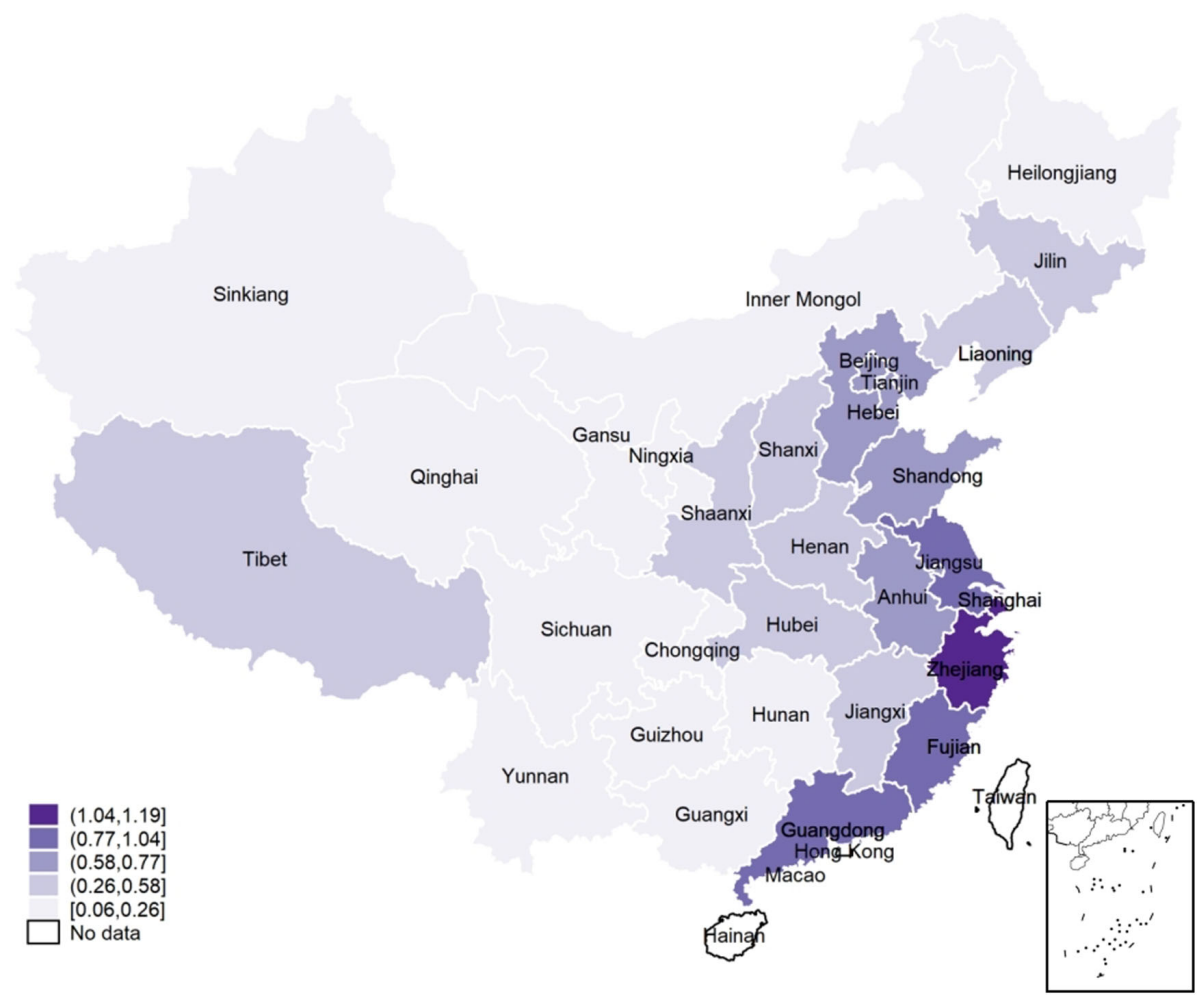
Distribution of Cultural Inclusion. The more inclusive areas correspond to darker purple.

We also used the share of cuisines other than local cuisine (e.g., in Beijing, the share of cuisines other than Beijing cuisine) as a proxy variable for cultural inclusion (*Out*_*cui*). Figure 2 shows the geographical distribution of cultural inclusion (*C*_*Inclu*), indicating that the geographical dimension of cultural inclusion is not evenly distributed, somehow proving that China is a good scenario to study cultural inclusion.

Our control-variables contained logarithmic firm size (*Size*), leverage (*LEV*), book-to-market ratio (*BM*), annual stock return (*RET*), analyst attention (*Attention*), turnover rate (*Turnover*), return on assets (*ROA*), shareholding ratio of top 10 shareholders(*Top_*10), largest shareholder holding (*Top_*1), the logarithm of management compensation (*Lncom*), board size (*Boardsize*), independent directors ratio (*Indeboard*), separation ratio of management & ownership (*Separation*), dual-role of board chairman (*CO*_*CEO*), dummy variable of state-owned firm (*SOE*), industry fixed (*Ind*) and year fixed-effects (*Year*). GDP growth rate (*GDP_growth*), GDP per-capita (*GDP_per*), logarithmic per-capita consumption (*Consume_per*) and population growth (*Pop_growth*) are used as regional control-indicators

Table 2 shows the data summary of our sample. On average, the cultural inclusion index *C_Inclu* equals 0.686 with a standard deviation of 0.3219, indicating significant differences between regions in terms of inclusion. In addition, the main dependent variable *TFP_lp* also exhibits significant variations across firms.

**Table 2:** Summary statistics.

Variables	Obs	Ave	Std	Med	Min	Max
TFP_LP	10140	8.282	0.998	8.174	6.209	10.809
C_Inclu	10140	0.686	0.319	0.754	0.065	1.191
Out_cui	10140	0.818	0.184	0.892	0.101	1.000
LEV	10140	0.399	0.192	0.394	0.050	0.823
Size	10140	22.247	1.277	22.049	20.079	26.207
ROA	10140	0.051	0.046	0.046	−0.155	0.187
BM	10140	0.600	0.235	0.596	0.143	1.135
Attention	10140	10.747	9.967	7.000	1.000	46.000
RET	10140	0.083	0.513	−0.054	−0.575	2.088
Turnover	10140	588.876	471.357	452.568	51.728	2688.277
TOP_1	10140	35.733	14.644	34.155	9.230	73.660
TOP_10	10140	60.110	14.253	61.090	25.140	90.560
SOE	10140	0.370	0.483	0.000	0.000	1.000
Boardsize	10140	8.764	1.730	9.000	5.000	15.000
Indeboard	10140	0.373	0.053	0.333	0.333	0.571
Separation	10140	4.617	7.528	0.000	0.000	28.283
Lncom	10140	15.512	0.671	15.477	13.846	17.332
CO_CEO	10140	0.274	0.446	0.000	0.000	1.000
GDP_per	10140	7.020	3.175	6.381	2.123	15.310
GDP_growth	10140	108.511	1.939	108.000	103.500	114.600
Pop_growth	10140	5.090	2.274	5.010	−0.440	10.840
Consume_per	10140	9.826	0.426	9.842	8.888	10.677

*Obs* observation, *std* standard deviation, *Ave* average, *Med* median.

## Empirical results

### Main regression

We used formula (9) to investigate the correlation between cultural inclusion and corporate sustainability:

9 $$TFP\_LP_{i,p,t} = \alpha + \beta C\_Inclu_{p,t} + \gamma Control_{i,p,t - 1} + {\it{\epsilon }}_{i,p,t}$$

Where, *TFP*_*LP*_*i,p,t*_ is *TFP* (measured by LP-semiparametric approach) of firm *i* situated in provincial unit *p* within year *t*. *C_Inclu*_*p,t*_ is the cultural inclusion index of provincial unit *p*, and *Control*_*i,p,t*-1_ are control-variables with one year lag. Year and industry fixed-effects are taken, and robust clustering criteria errors for firms and years are taken in our regression.

The results of our baseline regressions are given in Table 3, where regression (1) presents the baseline regression for all firms in our sample. Regression (3) further controls for geographical development indices (including *GDP*_*per*, *GDP*_*growth*, *Pop*_*growth*, and *Consume*_*per*). We can find the correlation of *C_Inclu* and *TFP_LP* is significantly positive at the 1% level with or without controlling for the geographical development index, which indicates that cultural inclusion can significantly contribute to sustainable corporate development.

**Table 3:** The impact of cultural inclusion on corporate sustainability.

TFP_LP	(1)	(2)	(3)	(4)
C_Inclu	0.140*** (8.253)		0.122*** (5.229)	
Out_cui		0.158*** (5.802)		0.155*** (5.127)
Size	0.536*** (66.381)	0.536*** (66.103)	0.536*** (66.069)	0.534*** (65.856)
ROA	2.765*** (17.671)	2.771*** (17.620)	2.763*** (17.592)	2.780*** (17.683)
LEV	1.017*** (25.984)	1.011*** (25.743)	1.014*** (25.828)	1.020*** (25.970)
BM	0.002 (0.053)	−0.004 (−0.102)	0.005 (0.134)	0.008 (0.207)
Attention	0.003*** (5.657)	0.003*** (5.134)	0.003*** (5.584)	0.003*** (5.331)
RET	0.064*** (4.643)	0.065*** (4.734)	0.064*** (4.645)	0.064*** (4.652)
Turnover	−0.000 (−0.918)	−0.000 (−0.989)	−0.000 (−1.008)	−0.000 (−1.057)
TOP_1	0.000 (0.390)	0.000 (0.392)	0.000 (0.421)	0.000 (0.288)
TOP_10	0.002*** (3.862)	0.002*** (4.245)	0.002*** (3.840)	0.002*** (3.912)
Boardsize	−0.011*** (−3.387)	−0.013*** (−3.986)	−0.012*** (−3.480)	−0.013*** (−3.764)
Indeboard	−0.184* (−1.778)	−0.218** (−2.101)	−0.193* (−1.863)	−0.217** (−2.088)
Lncom	0.048*** (5.208)	0.059*** (6.466)	0.046*** (4.937)	0.049*** (5.256)
SOE	0.073*** (5.354)	0.058*** (4.302)	0.074*** (5.382)	0.065*** (4.773)
Separation	0.001** (2.204)	0.001** (1.972)	0.002** (2.272)	0.002** (2.402)
CO_CEO	−0.038*** (−3.421)	−0.033*** (−2.994)	−0.037*** (−3.399)	−0.035*** (−3.223)
GDP_per			0.000 (0.884)	−0.000 (−0.479)
GDP_growth			0.001 (0.224)	0.007 (1.444)
Pop_growth			0.010*** (4.003)	0.013*** (5.248)
Consume_per			0.003 (0.074)	0.124*** (2.906)
Constant	−5.334*** (−28.552)	−5.512*** (−29.359)	−5.489*** (−6.741)	−7.308*** (−8.787)
Fixed-effect	√	√	√	√
Obs	10140	10140	10140	10140
*R*-squared	0.787	0.786	0.787	0.787

Significance: ***p < 0.01, **p < 0.05, *p < 0.1.

Regressions (2) and (4) examine correlation with cultural inclusion and corporate sustainability by using the proxy variable *Out_cui* for cultural inclusion. The results in columns (2) and (4) suggest that using the proxy variable *Out_cui* of inclusion does not change our main result: greater cultural inclusion is generally associated with higher total factor productivity. Overall, our regression results suggest a positive contribution of cultural inclusion to corporate sustainability.

### Endogenous issues

Although we explicitly controlled for as many variables as possible in the baseline regression, there are still potential endogenous issues. To address these concerns, we referred to Lei et al. (2022) to take the mountainous undulation of the region (*Moun_Undu*) measured by Feng et al. (2008) to conduct an instrumental variable test for cultural inclusion (Feng et al., 2008; Lei et al., 2022). *Moun_Undu* is highly correlated with cultural inclusion because complex interlocking mountainous terrain makes human interactions more difficult at the geographic level, and the precondition for inclusion is deeper human interactions (Feng et al., 2008). Second, more complex topography makes it easier for regions to be historically divided into small, relatively enclosed areas, which makes migration more difficult and the population more divided, thus making cross-regional cultural transmission difficult, and thus closed areas are more likely to develop xenophobic ideas. In general, higher mountain undulation may inhibit the formation of cultural inclusion, but mountain undulation is a purely geographic natural shock, which is the result of natural geographical evolution and cannot be influenced by firm sustainability; therefore, it is an exogenous enough instrumental variable for cultural inclusion (Lei et al., 2022).

Based on the mountain undulation, we used 2SLS method:

10 $$C\_Inclu_{p,t} = a + b \times Moun\_Undu_{p,t} + r \times Control_{i,p,t - 1} + \delta _{p,t}$$

11 $$TFP\_LP_{i,p,t} = \alpha + \beta \widehat {C\_Inclu}_{p,t} + \gamma Control_{i,p,t - 1} + {\it{\epsilon }}_{i,p,t}$$

Where, *Moun*_*Undu*_*p*_ is the mountain undulation of *p* province in *t* year of the first step regression, $\widehat {C\_inclu}_{p,t}$ is the fitted value of cultural inclusion from the first step, and other variables as before.

Our results of formula (9) and (10) are given in Table 4. We took the mountain undulation of regions (*Moun_Undu*) and reported our findings of IV regression in columns (1)-(2). We found that on the first step, higher mountain undulation *Moun_Undu* significantly reduced cultural inclusion, while on the second step, we found that the cultural inclusion $\widehat {C\_inclu}$ fitted with mountain undulation significantly enhanced corporate sustainability. In addition, two *F* statistics are all far larger than the 10% level critical value (16.38) of stock Yogo test, rejecting the original assumption of instrumental variable weak identification. Both *F* tests confirmed that *Moun_Undu* was not a weak instrumental variable of cultural inclusion.

**Table 4:** The impact of cultural inclusion on corporate sustainability: instrumental variable method.

TFP_LP	(1)	(2)	(3)	(4)
	Step 1	Step 2	Step 1	Step 2
	C_Inclu	TFP_LP	Out_cui	TFP_LP
Moun_Undu	−0.129*** (−45.240)		−0.119*** (−41.787)	
C_Inclu		0.352*** (6.131)		
Out_cui				0.382*** (6.099)
Size	−0.005* (−1.658)	0.544*** (66.726)	0.010*** (4.488)	0.538*** (65.806)
ROA	0.250*** (4.626)	2.712*** (16.752)	0.081** (2.168)	2.769*** (17.129)
LEV	0.038*** (2.715)	1.022*** (25.172)	−0.010 (−1.022)	1.039*** (25.521)
BM	0.023 (1.608)	−0.001 (−0.019)	−0.005 (−0.497)	0.009 (0.237)
Attention	−0.001*** (−3.404)	0.003*** (5.498)	0.000* (1.913)	0.003*** (4.827)
RET	0.008 (1.495)	0.062*** (4.357)	0.004 (0.999)	0.064*** (4.431)
Turnover	−0.000** (−2.183)	−0.000 (−0.549)	−0.000 (−0.985)	−0.000 (−0.746)
TOP_1	−0.000 (−1.280)	0.000 (0.499)	0.000* (1.750)	0.000 (0.153)
TOP_10	0.000** (2.013)	0.002*** (3.708)	0.000 (0.216)	0.002*** (3.922)
Boardsize	−0.005*** (−3.675)	−0.011*** (−3.062)	0.002* (1.686)	−0.013*** (−3.818)
Indeboard	−0.224*** (−5.737)	−0.142 (−1.321)	−0.022 (−0.740)	−0.213** (−1.976)
Lncom	0.004 (1.192)	0.049*** (5.161)	−0.017*** (−6.385)	0.057*** (5.930)
SOE	−0.071*** (−14.186)	0.089*** (6.101)	0.004 (1.038)	0.062*** (4.524)
Separation	0.000 (1.360)	0.002** (2.355)	−0.000 (−1.169)	0.002*** (2.686)
CO_CEO	0.007 (1.543)	−0.045*** (−3.968)	−0.009*** (−2.886)	−0.039*** (−3.472)
GDP_per	0.000*** (3.481)	−0.000 (−0.109)	0.000*** (29.855)	−0.000** (−2.565)
GDP_growth	0.020*** (7.996)	0.003 (0.625)	−0.021*** (−10.658)	0.018*** (3.313)
Pop_growth	0.017*** (16.734)	0.006** (2.181)	−0.007*** (−8.812)	0.014*** (5.662)
Consume_per	0.549*** (30.489)	−0.113** (−2.222)	−0.336*** (−25.159)	0.209*** (4.346)
Constant	−6.582*** (−16.679)	−5.003*** (−5.836)	6.159*** (19.738)	−9.671*** (−10.067)
Fixed-effect	√	√	√	√
Obs	10140	10140	10140	10140
*R*-squared	0.676	0.785	0.494	0.785
*F* statis: Cragg-Donald Wald	2360.165		3873.292	
*F* statis: Kleibergen-Paap rk Wald	2046.676		1746.114	

Significance: ***p < 0.01, **p < 0.05, *p < 0.1.

In columns (3) to (4), we used the alternative variable of cultural inclusion *Out_cui* to corroborate the authenticity of IV regression. Our results were the same as the above, and Both *F* tests showed that our instrumental variable *Moun_Undu* is not weak. In general, our findings suggest the explanation of the causality between cultural inclusion and corporate sustainability.

### Regression discontinuity design

In the previous section, we conducted IV regression using mountain undulation to mitigate the endogeneity issues, we now employ a regression discontinuity design (RDD) to mitigate the impacts of the bidirectional causality.

Specifically, Shanghai used to be part of Jiangsu Province. Shanghai and Jiangsu share similar dialects, clientele and practices. However, Shanghai is recognized as the most economically developed city in China and is the country’s international economic, financial, trade, shipping as well as technological innovation center. In addition, Shanghai is recognized as more culturally inclusive due to its important position as a transportation hub in China. Considering that Shanghai and Jiangsu are bordering each other, we chose Shanghai and Jiangsu for the RDD test.

We constructed the dummy variable *RDD_SJ* and used it as the independent variable. When the firm is located in Shanghai, *RDD_SJ* is equal to 1; when it is located in Jiangsu, *RDD_SJ* is equal to 0. Our regression results are presented in Table 5, with column (1) controlling for year and industry fixed-effects and column (2) controlling for region fixed-effect. The results are as expected, with better corporate sustainability in Shanghai. In addition, we also used Shanghai and Zhejiang for RDD testing for similar reasons as above. We constructed the dummy variable *RDD_SZ* as above, and the similar empirical results are shown in columns (3) and (4) of Table 5.

**Table 5:** Regression discontinuity design.

TFP_LP	(1)	(2)	(3)	(4)
RDD_SJ	0.111*** (4.336)	0.103*** (3.914)		
RDD_SZ			0.086*** (3.395)	0.116*** (4.195)
Size	0.590*** (33.953)	0.534*** (29.844)	0.525*** (30.346)	0.484*** (30.338)
ROA	2.795*** (8.229)	3.672*** (9.606)	2.831*** (8.271)	3.558*** (9.121)
LEV	0.980*** (9.627)	1.300*** (14.012)	1.185*** (12.501)	1.330*** (14.449)
BM	−0.142* (−1.870)	0.059 (0.771)	0.150* (1.887)	0.345*** (4.643)
Attention	−0.002 (−1.039)	−0.001 (−0.441)	0.003** (2.328)	0.002* (1.769)
RET	−0.014 (−0.458)	−0.028 (−1.023)	−0.005 (−0.191)	0.015 (0.662)
Turnover	0.000 (1.159)	−0.000 (−0.088)	0.000 (0.200)	−0.000 (−1.137)
TOP_1	0.003*** (3.048)	0.003*** (2.764)	0.003*** (3.086)	0.004*** (3.460)
TOP_10	−0.001 (−1.153)	−0.004*** (−3.365)	−0.001 (−0.833)	−0.003*** (−2.966)
Boardsize	0.004 (0.403)	−0.031*** (−2.625)	−0.017* (−1.906)	−0.021* (−1.958)
Indeboard	0.652*** (2.700)	−0.381 (−1.315)	−0.556** (−2.337)	−0.780*** (−2.809)
Lncom	0.082*** (3.964)	0.058** (2.470)	0.064*** (3.106)	0.056*** (2.630)
SOE	0.013 (0.335)	−0.042 (−1.092)	−0.022 (−0.581)	−0.072* (−1.810)
Separation	0.001 (0.542)	0.002 (1.490)	−0.001 (−1.040)	−0.002 (−1.283)
CO_CEO	−0.097*** (−4.455)	−0.115*** (−4.444)	−0.064*** (−3.043)	−0.057** (−2.381)
Constant	−7.276*** (−17.014)	−4.694*** (−10.880)	−4.648*** (−11.679)	−3.773*** (−8.915)
Year-Ind FE	√	×	√	×
Region FE	×	√	×	√
Obs	2083	2083	2264	2264
*R*-squared	0.836	0.729	0.818	0.711

Significance: ***p < 0.01, **p < 0.05, *p < 0.1.

### Robustness test: replacement of independent variable

An important basis for differentiating the taste of a cuisine is its spiciness, which is particularly prominent among the differences in food cultures. Therefore, in this section, we used the degree of deviation of the quantitative value of spiciness from the spiciness of the local cuisine, *Dspicy*, as a proxy for cultural inclusion (Batra et al., 2017; Byrnes and Hayes, 2013; Byrnes and Hayes, 2016; Chang et al., 2010; Li et al., 2021; Ludy and Mattes, 2012; Sun et al., 2022). Our regressions are presented in Table 6, where we can see that the positive relevance between cultural inclusion and corporate *TFP* is robust.

**Table 6:** The impact of cultural inclusion on corporate sustainability (replacement of independent variable).

TFP_LP	(1)	(2)
Dspicy	0.021*** (7.491)	0.019*** (4.726)
Size	0.541*** (66.095)	0.540*** (65.977)
ROA	2.780*** (17.274)	2.777*** (17.189)
LEV	1.035*** (25.359)	1.031*** (25.256)
BM	0.010 (0.245)	0.013 (0.335)
Attention	0.003*** (5.386)	0.003*** (5.298)
RET	0.067*** (4.649)	0.067*** (4.643)
Turnover	−0.000 (−0.843)	−0.000 (−0.933)
TOP_1	0.000 (0.368)	0.000 (0.405)
TOP_10	0.002*** (3.970)	0.002*** (3.921)
Boardsize	−0.012*** (−3.385)	−0.012*** (−3.471)
Indeboard	−0.193* (−1.801)	−0.201* (−1.879)
Lncom	0.053*** (5.641)	0.050*** (5.307)
SOE	0.068*** (4.851)	0.069*** (4.950)
Separation	0.002** (2.269)	0.002** (2.362)
CO_CEO	−0.040*** (−3.588)	−0.040*** (−3.583)
GDP_per		0.000 (1.225)
GDP_growth		0.001 (0.146)
Pop_growth		0.012*** (4.817)
Consume_per		−0.006 (−0.132)
Constant	−5.512*** (−28.536)	−5.552*** (−6.641)
Fixed-effect	√	√
Obs	10140	10140
*R*-squared	0.784	0.785

Significance: ***p < 0.01, **p < 0.05, *p < 0.1.

### Robustness test: replacement of dependent variable

We used the OP-semiparametric approach to calculate total factor productivity of firms (*TFP_OP*) as a robustness test. We used the three cultural inclusion indices mentioned above as independent variables. Columns (1) to (3) in Table 7 present our results with controlling firm characteristics, while columns (4) to (6) further control geographical development characteristics. In all regressions, we found the impact of cultural inclusion remains robust.

**Table 7:** The impact of cultural inclusion on corporate sustainability (replacement of dependent variable).

TFP_OP	(1)	(2)	(3)	(4)	(5)	(6)
C_Inclu	0.093*** (5.813)			0.101*** (4.515)		
Out_cui		0.104*** (4.198)			0.097*** (3.459)	
Dspicy			0.012*** (4.595)			0.012*** (3.175)
Size	0.225*** (29.604)	0.225*** (29.558)	0.225*** (29.578)	0.225*** (29.460)	0.224*** (29.313)	0.225*** (29.409)
ROA	2.192*** (15.548)	2.196*** (15.528)	2.192*** (15.532)	2.184*** (15.446)	2.201*** (15.576)	2.189*** (15.459)
LEV	0.724*** (19.502)	0.720*** (19.355)	0.722*** (19.418)	0.720*** (19.313)	0.725*** (19.433)	0.721*** (19.327)
BM	0.172*** (4.731)	0.168*** (4.610)	0.172*** (4.715)	0.170*** (4.653)	0.173*** (4.746)	0.171*** (4.687)
Attention	0.000 (0.563)	0.000 (0.188)	0.000 (0.560)	0.000 (0.500)	0.000 (0.299)	0.000 (0.511)
RET	0.072*** (5.512)	0.073*** (5.584)	0.072*** (5.551)	0.072*** (5.519)	0.072*** (5.557)	0.072*** (5.569)
Turnover	0.000 (1.175)	0.000 (1.118)	0.000 (1.120)	0.000 (1.137)	0.000 (1.066)	0.000 (1.066)
TOP_1	−0.001 (−1.496)	−0.001 (−1.490)	−0.001 (−1.476)	−0.001 (−1.417)	−0.001 (−1.516)	−0.001 (−1.415)
TOP_10	0.000 (0.471)	0.000 (0.745)	0.000 (0.571)	0.000 (0.489)	0.000 (0.566)	0.000 (0.537)
Boardsize	−0.013*** (−3.896)	−0.014*** (−4.317)	−0.013*** (−3.991)	−0.013*** (−3.939)	−0.013*** (−4.178)	−0.013*** (−3.999)
Indeboard	−0.356*** (−3.637)	−0.379*** (−3.869)	−0.363*** (−3.705)	−0.354*** (−3.608)	−0.375*** (−3.826)	−0.361*** (−3.682)
Lncom	−0.090*** (−10.050)	−0.082*** (−9.336)	−0.088*** (−9.884)	−0.090*** (−10.001)	−0.088*** (−9.776)	−0.089*** (−9.961)
Separation	−0.038*** (−2.975)	−0.049*** (−3.777)	−0.042*** (−3.220)	−0.037*** (−2.891)	−0.045*** (−3.514)	−0.041*** (−3.133)
CO_CEO	0.001** (2.054)	0.001* (1.891)	0.001* (1.955)	0.001** (1.989)	0.001** (2.078)	0.001* (1.950)
SOE	−0.023** (−2.119)	−0.019* (−1.829)	−0.022** (−2.035)	−0.022** (−2.092)	−0.021* (−1.938)	−0.022** (−2.030)
GDP_percapita				0.000 (0.819)	0.000 (0.039)	0.000 (1.070)
GDP_growth				−0.007 (−1.526)	−0.003 (−0.698)	−0.008* (−1.803)
Pop_growth				0.005** (2.073)	0.007*** (3.104)	0.007*** (2.722)
Consume_percapita				−0.046 (−1.128)	0.041 (0.988)	−0.038 (−0.924)
Constant	−0.592*** (−3.422)	−0.709*** (−4.113)	−0.589*** (−3.403)	0.579 (0.748)	−0.649 (−0.812)	0.677 (0.864)
Fixed-effect	√	√	√	√	√	√
Obs	10140	10140	10140	10140	10140	10140
*R*-squared	0.549	0.548	0.549	0.550	0.549	0.549

Significance: ***p < 0.01, **p < 0.05, *p < 0.1.

## Further analysis

### Transmission of cultural inclusion in society

In the previous chapter, we demonstrated the impact of cultural inclusion on corporate sustainability. Now we explore how cultural inclusion spreads through society. Many studies have shown that culture has a lasting impact on humans even when they migrate to different countries (Guiso et al., 2006; Fisman and Miguel, 2007; DeBacker et al., 2015; Liu, 2015). If this is the case, we would expect top managers to bring cultural values from their home regions and to help disseminate these values through corporate leadership. We can further identify the causal impact of cultural inclusion along the lines of the above literature - in addition to the impact identified using the IV approach.

More specifically, we executed two tests to validate this transmission path. In the first test, we examined whether the cultural characteristics carried by a firm’s CEO affect corporate sustainability. We performed a regression analysis using the culturally inclusive characteristics of the CEO’s birthplace (*Birth_Inclu*) as the independent variable to examine whether top managers’ cultural characteristics can play a significant role in corporate sustainability.

The results in Table 8 indicate (columns (1) and (2), where column (1) controls for firm characteristics and column (2) further controls for regional characteristics) that there is a significant positive relationship between cultural inclusion in the CEO’s birthplace and corporate sustainability. Top managers may have decision-making power in the firm, and the cultural characteristics they themselves carry can cause systematic biases in their individual perceptions, which in turn cause differences in corporate sustainability outcomes. This suggested that top managers play an important role in the transmission of sociocultural inclusion.

**Table 8:** The role of corporate top managers in transmitting cultural inclusion.

TFP_LP	(1)	(2)	(3)	(4)
Birth_Inclu	0.080** (2.419)	0.104*** (2.904)		
More_Inclu			0.054* (1.749)	0.059* (1.870)
Size	0.537*** (32.608)	0.543*** (32.306)	0.536*** (32.514)	0.538*** (32.100)
ROA	2.913*** (9.753)	2.896*** (9.710)	2.951*** (9.876)	2.958*** (9.913)
LEV	1.109*** (13.330)	1.093*** (13.115)	1.106*** (13.205)	1.100*** (13.108)
BM	0.151* (1.937)	0.132* (1.687)	0.157** (2.023)	0.149* (1.906)
Attention	0.004*** (3.024)	0.003*** (2.754)	0.003*** (2.763)	0.003*** (2.633)
RET	0.107*** (3.750)	0.105*** (3.716)	0.112*** (3.951)	0.112*** (3.954)
Turnover	−0.000*** (−3.087)	−0.000*** (−2.980)	−0.000*** (−3.320)	−0.000*** (−3.323)
TOP_1	0.001 (1.318)	0.001 (1.414)	0.001 (1.384)	0.001 (1.460)
TOP_10	0.000 (0.484)	0.001 (0.593)	0.000 (0.474)	0.000 (0.466)
Boardsize	−0.028*** (−3.809)	−0.029*** (−3.987)	−0.029*** (−3.947)	−0.029*** (−3.969)
Indeboard	−0.401* (−1.713)	−0.433* (−1.838)	−0.438* (−1.871)	−0.459* (−1.949)
Lncom	0.054*** (2.946)	0.055*** (2.993)	0.059*** (3.300)	0.057*** (3.124)
SOE	0.059* (1.817)	0.056* (1.740)	0.049 (1.502)	0.050 (1.547)
Separation	0.000 (0.239)	−0.000 (−0.049)	0.000 (0.185)	0.000 (0.085)
CO_CEO	−0.022 (−1.000)	−0.023 (−1.031)	−0.019 (−0.840)	−0.019 (−0.848)
GDP_per		0.000 (1.439)		0.000 (1.472)
GDP_growth		−0.015 (−1.376)		−0.015 (−1.410)
Pop_growth		0.008 (1.272)		0.009 (1.518)
Consume_per		−0.190** (−1.990)		−0.137 (−1.431)
Constant	−5.080*** (−15.022)	−1.892 (−1.050)	−5.119*** (−15.065)	−2.255 (−1.238)
Fixed-effect	√	√	√	√
Obs	2132	2132	2132	2132
*R*-squared	0.841	0.841	0.841	0.841

Significance: ***p < 0.01, **p < 0.05, *p < 0.1.

In the second test, we constructed the dummy variable *More_Inclu*. *More_Inclu* equals 1 when a firm’s CEO is from a region that is more culturally inclusive than the region where his firm is located, and 0 otherwise. We set this dummy variable as the independent variable to examine whether managers can have a transmission effect on cultural inclusion. Columns (3) and (4) of Table 8 showed that a strong climate of cultural inclusion in the CEO’s birthplace can strengthen the link between cultural inclusion and corporate sustainability. The possible reason for this is that a stronger cultural inclusion in the birthplace means that CEOs are likely to be more influenced by cultural inclusion and develop their personalities accordingly. As CEOs have an important role in influencing important business decisions, they have the willingness and ability to use their position, power and influence to provide a supportive environment for corporate sustainability. Therefore, the cultural background of CEOs enhances the impact of cultural inclusion on corporate sustainability and enhances the positive transmission of cultural inclusion in corporate behavioral decisions.

### Mechanism of cultural inclusion

We investigate the mechanism by which cultural inclusion promotes corporate sustainability in this section. For firms, human capital is their core competency, and their sustainable development is inextricably linked to the accumulation of their human capital (Chen et al., 2021). A large body of research evidence suggests that staff’ perception of organizational characteristics is an important factor in motivating their organizational citizenship behavior. The more staff feel cared for by the organization, the more they will demonstrate organizational citizenship and thus contribute to the sustainable development of the firm (Hancock et al., 2013; Paillé et al., 2014). Management will be influenced by the local culture and will show distinct cultural characteristics in their thoughts and actions, which will be reflected in corporate behavior such as organizational planning and governance decisions (Chen et al., 2014). Based on this, we believe that an inclusive external environment can lead firms to increase the level of inclusion and care for their staff, which we call the “humanistic care hypothesis”.

We used *CSR_emp* disclosed by Hexun (https://www.hexun.com/), a segmentation indicator reflecting CSR toward staff, as a measure for extent to which firms care for their staff. We took the 2SLS method to conduct mechanism tests to validate the mechanisms of corporate care for staff (Griffin et al., 2021). In the first step we regressed the core explanatory variable (*C_Inclu*) using the mediating variable (*CSR_staff*) and calculated the fitted values of the mediating variable based on the regression coefficients. We regressed these fitted values using explanatory variable (*TFP_LP*) in the second step, and our empirical results are presented in Table 9.

**Table 9:** The impact of cultural inclusion on corporate sustainability: mechanism.

	(1)	(2)	(3)	(4)	(5)	(6)
	Step 1	Step 2	Step 1	Step 2	Step 1	Step 2
	CSR_staff	TFP_LP	CSR_staff	TFP_LP	CSR_staff	TFP_LP
C_Inclu	0.589*** (4.145)					
Out_cui			0.448** (2.429)			
Dspicy					0.091*** (3.954)	
CSR_staff		0.236*** (3.402)		0.347** (2.189)		0.205*** (3.070)
Size	0.781*** (15.041)	0.357*** (6.444)	0.772*** (14.871)	0.271** (2.187)	0.779*** (15.016)	0.381*** (7.150)
ROA	2.808*** (3.458)	2.111*** (6.591)	2.926*** (3.600)	1.782*** (3.119)	2.795*** (3.440)	2.204*** (7.246)
LEV	−0.078 (−0.371)	1.049*** (16.524)	−0.051 (−0.240)	1.055*** (12.676)	−0.080 (−0.380)	1.048*** (17.838)
BM	−2.313*** (−10.497)	0.558*** (3.290)	−2.290*** (−10.395)	0.810** (2.178)	−2.313*** (−10.500)	0.488*** (2.994)
Attention	0.003 (0.714)	0.003** (2.493)	0.002 (0.543)	0.002* (1.659)	0.003 (0.764)	0.003*** (2.800)
RET	−0.428*** (−5.001)	0.167*** (4.404)	−0.424*** (−4.942)	0.213*** (2.874)	−0.426*** (−4.978)	0.154*** (4.303)
Turnover	−0.000 (−0.023)	−0.000 (−0.521)	−0.000 (−0.114)	−0.000 (−0.344)	−0.000 (−0.071)	−0.000 (−0.586)
TOP_1	−0.004 (−1.444)	0.001 (1.357)	−0.004 (−1.527)	0.002 (1.257)	−0.004 (−1.426)	0.001 (1.304)
TOP_10	−0.002 (−0.764)	0.002*** (2.822)	−0.002 (−0.691)	0.002** (2.268)	−0.002 (−0.733)	0.002*** (3.000)
Boardsize	−0.026 (−1.067)	−0.006 (−0.791)	−0.030 (−1.228)	−0.002 (−0.234)	−0.027 (−1.083)	−0.007 (−1.005)
Indeboard	1.243* (1.902)	−0.489** (−2.451)	1.116* (1.708)	−0.610** (−2.020)	1.234* (1.889)	−0.455** (−2.476)
Lncom	0.592*** (10.573)	−0.090** (−2.021)	0.602*** (10.729)	−0.156 (−1.611)	0.594*** (10.607)	−0.071* (−1.673)
SOE	0.783*** (9.533)	−0.112** (−1.985)	0.738*** (9.055)	−0.194 (−1.619)	0.774*** (9.454)	−0.089* (−1.656)
Separation	0.008** (2.002)	−0.000 (−0.213)	0.008** (2.072)	−0.001 (−0.573)	0.008* (1.944)	−0.000 (−0.020)
CO_CEO	−0.049 (−0.788)	−0.029 (−1.622)	−0.039 (−0.632)	−0.025 (−1.025)	−0.047 (−0.761)	−0.031* (−1.835)
GDP_per	0.000 (0.208)	0.000 (0.435)	−0.000 (−0.293)	0.000 (0.119)	0.000 (0.368)	0.000 (0.556)
GDP_growth	−0.040 (−1.192)	0.012 (1.267)	−0.023 (−0.660)	0.017 (1.173)	−0.050 (−1.478)	0.011 (1.250)
Pop_growth	0.048*** (3.380)	−0.001 (−0.162)	0.061*** (4.476)	−0.008 (−0.675)	0.055*** (4.024)	0.001 (0.163)
Consume_per	−0.130 (−0.484)	0.023 (0.305)	0.333 (1.227)	0.005 (0.053)	−0.164 (−0.601)	0.028 (0.405)
Constant	−15.510*** (−2.838)	−2.125 (−1.106)	−21.633*** (−3.760)	−0.173 (−0.051)	−14.040** (−2.543)	−2.672 (−1.485)
Fixed-effect	√	√	√	√	√	√
Obs	10140	10140	10140	10140	10140	10140
*R*-squared	0.292	0.785	0.291	0.785	0.292	0.785

Significance: ***p < 0.01, **p < 0.05, *p < 0.1.

Regressions (1)-(2) in Table 9 show our mechanism test of the humanistic care hypothesis that cultural inclusion affects the corporate sustainability. In columns (1) to (2), we set *CSR_emp*, the corporate social responsibility to staff, as a mediating variable. We found that in the first step, higher cultural inclusion increases social responsibility towards staff. In the second step, we found that *CSR_emp* fitted with cultural inclusion increases total factor productivity, suggesting that cultural inclusion serves to promote sustainable corporate development through the mechanism of increasing corporate care for staff. The empirical results in Table 9 validated our proposed “humanistic care hypothesis” of cultural inclusion. We further validated the robustness of the humanistic care hypothesis by replacing *C_Inclu* in columns (3)-(6) with two alternative variables of cultural inclusion (*Out_cui* and *Dspicy*).

### Cross-sectional inspection

Cultural inclusion may have heterogeneous effects across firms, and the cost of sustainable development varies. This section explores the heterogeneity of the influence of cultural inclusion on corporate sustainability from two perspectives: industry competitiveness and analyst attention.

The degree of industry competition refers to the intensity of competition between companies in their industries (Jaworski and Kohli, 1993). It stems from the emergence of competitors or the lack of future development opportunities that lead to competition for resources between companies, such as price wars, promotional wars, additional services (Martin and Javalgi, 2016), etc. In a more competitive industry, the centripetal force of the firm becomes more important. Cultural inclusion gives firms a humanistic and caring spirit, which may lead to firms giving more tolerance and care to their staff, forming a strong corporate centripetal force, and thus creating a competitive advantage for firms in the face of a competitive external environment. Thus, cultural inclusion may have heterogeneous effects in industries with different levels of competition. We used the industry’s Herfindahl index (*Hhi*) to measure the industry’s degree of competition and constructed the dummy variable *Com* using the median of *Hhi*, which was included in the baseline model with the interaction term (*C_Inclu_Com*) of cultural inclusion (*C_Inclu*). Our empirical results are shown in (1) and (2) of Table 10, where the impact of cultural inclusion is more obvious in more intense degrees of competition industries.

**Table 10:** The impact of cultural inclusion on corporate sustainability: cross-sectional inspection.

TFP_LP	(1)	(2)	(3)	(4)
C_Inclu	0.117*** (5.128)	0.106*** (3.787)	0.114*** (6.050)	0.105*** (4.172)
C_Inclu _Com	0.059* (1.830)	0.063* (1.943)		
C_Inclu _Atten			0.063*** (4.392)	0.063*** (4.421)
Com	−0.411*** (−5.084)	−0.401*** (−5.009)		
Size	0.540*** (66.110)	0.541*** (65.933)	0.540*** (66.179)	0.541*** (66.027)
ROA	2.791*** (17.363)	2.784*** (17.265)	2.768*** (17.264)	2.761*** (17.165)
LEV	1.037*** (25.492)	1.031*** (25.271)	1.033*** (25.422)	1.026*** (25.210)
BM	0.009 (0.217)	0.011 (0.273)	0.014 (0.342)	0.016 (0.399)
Attention	0.003*** (5.401)	0.003*** (5.305)	0.003*** (3.917)	0.002*** (3.809)
RET	0.066*** (4.590)	0.066*** (4.584)	0.064*** (4.500)	0.064*** (4.493)
Turnover	−0.000 (−0.637)	−0.000 (−0.721)	−0.000 (−0.476)	−0.000 (−0.556)
TOP_1	0.000 (0.387)	0.000 (0.438)	0.000 (0.472)	0.000 (0.521)
TOP_10	0.002*** (3.784)	0.002*** (3.782)	0.002*** (3.717)	0.002*** (3.719)
Boardsize	−0.011*** (−3.234)	−0.012*** (−3.355)	−0.011*** (−3.237)	−0.012*** (−3.365)
Indeboard	−0.187* (−1.744)	−0.196* (−1.826)	−0.192* (−1.797)	−0.202* (−1.882)
Lncom	0.052*** (5.469)	0.050*** (5.266)	0.051*** (5.388)	0.049*** (5.192)
SOE	0.072*** (5.128)	0.073*** (5.175)	0.073*** (5.226)	0.074*** (5.273)
Separation	0.002** (2.446)	0.002** (2.490)	0.002** (2.424)	0.002** (2.464)
CO_CEO	−0.041*** (−3.669)	−0.041*** (−3.637)	−0.041*** (−3.658)	−0.041*** (−3.625)
GDP_per		0.000 (1.031)		0.000 (1.068)
GDP_growth		0.002 (0.481)		0.003 (0.570)
Pop_growth		0.011*** (4.137)		0.010*** (4.076)
Consume_per		−0.011 (−0.263)		−0.013 (−0.302)
Constant	−5.132*** (−28.978)	−5.333*** (−6.504)	−5.502*** (−28.578)	−5.730*** (−6.956)
Fixed-effect	√	√	√	√
Obs	10140	10140	10140	10140
*R*-squared	0.784	0.785	0.785	0.785

Significance: ***p < 0.01, **p < 0.05, *p < 0.1.

Numerous research suggests analysts play an informational and external regulatory role, especially in Chinese listed firms with imperfect governance structure and generally low information transparency (Dong et al., 2021). The cultural inclusion may have a heterogeneous impact on firms with different levels of analyst attention. On the one hand, based on the information interpretation and messaging role of analysts, firms may be more motivated to engage in more humanistic behavior to develop a good corporate reputation by conveying positive corporate messages to the market through analysts (Ioannou and Serafeim, 2015). In addition, because of the external regulatory role of analysts, analysts may be influenced by local cultural inclusion when conducting field research on firms, and firms are required to engage in more humanistic activities (Dhaliwal et al., 2012). Therefore, this paper argues that the humanistic care hypothesis formed by cultural inclusion has a stronger impact on firms with higher levels of analyst attention. We constructed the dummy variable *atten* using the median of *Attention*, which is included in the baseline model with the interaction term (*C_Inclu_att*) of cultural inclusion (*C_Inclu*). The coefficients of the interaction-item *C_Inclu_att* in columns (3)-(4) is significantly positive, indicating that humanistic care is more evident in firms with higher analyst attention. Specifically, the humanistic effect of firms with high analyst attention in regions with more inclusive external environment is stronger.

## Conclusion and implications

### Conclusion

We investigated how cultural inclusion contributes to corporate sustainability. Owing to the significant geographical differences in Chinese food culture, we used text analysis to quantify food taste and constructed a cultural inclusion index. We found significant differences in cultural inclusion among Chinese provinces. Our core finding is that firms in more culturally inclusive regions tend to exhibit more sustainable growth. These findings remained robust after a series of robustness tests.

We addressed potential endogeneity using an instrumental variable of geographic shock (i.e., mountainous undulation) to show a causality between cultural inclusion and corporate sustainability. We also conducted a regression discontinuity design (RDD) to mitigate the effects of bidirectional causality. In addition, our results suggested that top managers can use their corporate leadership to facilitate the transmission of cultural inclusion in society. Besides, we verified that cultural inclusion promotes corporate sustainability by improving humanistic care for employees, validating our “humanistic care hypothesis” associated with cultural inclusion. We also demonstrated that cultural inclusion has a heterogeneous impact on firms with different levels of industry competition and analyst attention.

Overall, our findings provided new empirical evidence on how informal institutions affect corporate sustainability. To our knowledge, our research might be the first to suggest that cultural inclusion affects firm sustainability. We confirmed the significant impact of cultural inclusion and demonstrate the importance of culture in the corporate sustainability process.

### Implications, suggestions, and limitations

Our paper mainly revealed how social norms affect corporate sustainability. Our study is most directly related to Lei et al. (2022). Lei et al. (2022) used the number of dialects in a region as a measure of cultural diversity because sociology considers language to be social and cognitive. Their study concluded that cultural diversity can influence corporate tax avoidance behavior, exposing the impact of regional culture on corporate governance. Similarly, based on the natural, social and cultural attributes of food, we used food culture as a representative of cultural inclusion. Our basic argument is that cultural inclusion significantly enhances the corporate total factor productivity and contributes to corporate sustainability. Our research showed that top managers played a crucial role in the transmission of cultural inclusion in society. In addition, cultural inclusion played a catalytic role in enhancing corporate humanism toward stakeholders. We summarized the mechanisms and transmission paths of cultural inclusion by relying on institutional theory. We explored how cultural inclusion leads firms to care their stakeholders more. Our study is an exploration of informal institutional impact research based on cultural inclusion, an important geoculture.

To our knowledge, our research may suggest for the first time that cultural inclusion is an important social norm for corporate sustainability and therefore may be an important reference for internal corporate governance, external investors and governments. Our study recommends that corporate managers develop more inclusive management systems. This is because our results show that firms giving more humanistic care to their stakeholders (especially employees) can promote corporate sustainability. Similarly, external investors can assess local levels of corporate sustainability by referring to local levels of cultural inclusion, thus making more informed investment decisions. Our results showed that corporate sustainability in less culturally inclusive regions needs further improvement. Therefore, we suggest that the government can develop relevant inclusive policies to guide firms in these regions to be more humane to their stakeholders. We hope that these inclusive policies will promote corporate sustainability and ultimately social sustainability. In addition, the government can give more resources to companies with high analyst attention and those with high industry competition. As these firms have stronger sustainable development ability, they are more likely to drive the social sustainability with their own sustainable development after acquiring sufficient resources.

We acknowledge that there are some limitations to our study of cultural inclusion. First, our cultural inclusion index was constructed using data from a single year. Although the literature has demonstrated the feasibility of this operational approach (Lei et al., 2022; Zhang, 2019), it is true that a continuous portrayal of cultural inclusion across regions in different years might yield richer findings. Second, our study is based entirely on a Chinese sample, a research design that has the advantage that the macro political institution is automatically controlled. And China, as a country with an unusually rich geoculture, provides a good research scenario for cultural inclusion. However, Shanghai, as the most culturally inclusive region in China, may have reached a moderate level of cultural inclusion. This makes it impossible to explore whether excessive cultural inclusion would negatively affect the corporate sustainability. In the future, if there are ways to address the strong endogeneity problem in cross-national studies, perhaps we can expand the sample to the whole world to explore the possible non-linear relationship between cultural inclusion and corporate sustainability.

## Data Availability

The data presented in this study are available on request from the corresponding author.
